# Effect of the Natural Sweetener Xylitol on Gut Hormone Secretion and Gastric Emptying in Humans: A Pilot Dose-Ranging Study

**DOI:** 10.3390/nu13010174

**Published:** 2021-01-08

**Authors:** Anne Christin Meyer-Gerspach, Jürgen Drewe, Wout Verbeure, Carel W. le Roux, Ludmilla Dellatorre-Teixeira, Jens F. Rehfeld, Jens J. Holst, Bolette Hartmann, Jan Tack, Ralph Peterli, Christoph Beglinger, Bettina K. Wölnerhanssen

**Affiliations:** 1 St. Clara Research Ltd. at St. Claraspital, 4002 Basel, Switzerland; christoph.beglinger@unibas.ch; 2 Faculty of Medicine, University of Basel, 4001 Basel, Switzerland; 3 Department of Clinical Pharmacology and Toxicology, University Hospital of Basel, 4001 Basel, Switzerland; juergen.drewe@unibas.ch; 4 Translational Research Center for Gastrointestinal Disorders, Catholic University of Leuven, 3000 Leuven, Belgium; wout.verbeure@kuleuven.be (W.V.); jan.tack@kuleuven.be (J.T.); 5 Diabetes Complications Research Centre, Conway Institute University College Dublin, 3444 Dublin, Ireland; carel.leroux@ucd.ie (C.W.l.R.); ludmilla.pessanha@ucd.ie (L.D.-T.); 6 Department of Clinical Biochemistry, Rigshospitalet, University of Copenhagen, 2100 Copenhagen, Denmark; Jens.F.Rehfeld@regionh.dk; 7 Department of Biomedical Sciences and Novo Nordisk Foundation Center for Basic Metabolic Research, Faculty of Health and Medical Sciences, University of Copenhagen, 2200 Copenhagen, Denmark; jjholst@sund.ku.dk (J.J.H.); bhartmann@sund.ku.dk (B.H.); 8 Department of Surgery, Clarunis, St. Claraspital, 4002 Basel, Switzerland; ralph.peterli@clarunis.ch

**Keywords:** xylitol, natural sweeteners, gut hormones, appetite-related sensations, gastric emptying, gastrointestinal symptoms, blood lipids, uric acid

## Abstract

Sugar consumption is associated with a whole range of negative health effects and should be reduced and the natural sweetener xylitol might be helpful in achieving this goal. The present study was conducted as a randomized, placebo-controlled, double-blind, cross-over trial. Twelve healthy, lean volunteers received intragastric solutions with 7, 17 or 35 g xylitol or tap water on four separate days. We examined effects on: gut hormones, glucose, insulin, glucagon, uric acid, lipid profile, as well as gastric emptying rates, appetite-related sensations and gastrointestinal symptoms. We found: (i) a dose-dependent stimulation of cholecystokinin (CCK), active glucagon-like peptide-1 (aGLP-1), peptide tyrosine tyrosine (PYY)-release, and decelerated gastric emptying rates, (ii) a dose-dependent increase in blood glucose and insulin, (iii) no effect on motilin, glucagon, or glucose-dependent insulinotropic peptide (GIP)-release, (iv) no effect on blood lipids, but a rise in uric acid, and (v) increased bowel sounds as only side effects. In conclusion, low doses of xylitol stimulate the secretion of gut hormones and induce a deceleration in gastric emptying rates. There is no effect on blood lipids and only little effect on plasma glucose and insulin. This combination of properties (low-glycemic sweetener which stimulates satiation hormone release) makes xylitol an attractive candidate for sugar replacement.

## 1. Introduction

Sugar consumption should be reduced in the general population according to WHO guidelines [1]. Individuals with diabetes and obesity are recommended a sugar-free, low glycemic diet. In this context, sugar substitutes, such as low-calorie sweeteners (LCS), might be helpful. However, a broad variety of substances are used and there is increasing evidence that the physiological effect of each substance on the human body has to be studied separately rather than evaluating them as a group, as sweeteners differ considerably in their chemical structure and their physiological properties, e.g., effects on taste perception, release of gut hormones, influence on gut microbiota composition, and stimulation of brain reward regions [2].

Xylitol is a simple alditol that is naturally occurring in various organisms and that has a long history of use in a wide variety of foods and dental products. One gram of xylitol provides around 2.4 kcal (compared to sucrose: 4 kcal/g), however xylitol is incompletely absorbed, and the energy available to the human body is even lower. The major part passes through the small intestine and is fermented by bacteria in the colon. As xylitol is increasingly used as a sugar substitute, questions arise regarding metabolic effects in humans, such as for instance release of gut hormones and satiating potential. A few studies have been carried out in this context in the late 1980s. In 1987, Shafer et al. showed that xylitol as part of a solid-food complex meal was able to decelerate gastric emptying measured by scintigraphy [3] which indicates that xylitol impacts the satiation system. In a trial published in 1989 by Salminen et al., oral administration of 30 g xylitol in 200 mL water compared to 30 g glucose in 200 mL water also resulted in a deceleration in gastric emptying [4]. Our own study group recently compared effects of acute intake of 75 g glucose, 50 g xylitol or 75 g erythritol in 300 mL water versus placebo (tap water) on gut hormones and gastric emptying. Fifty grams xylitol induced a significant retardation in gastric emptying (GE), and led to a marked increase in plasma cholecystokinin (CCK) and active glucagon-like-peptide 1 (aGLP-1) concentrations [5]. Potential side-effects known so far include osmotic effects that can cause discomfort in some individuals in case of rapid overconsumption. The load used in our preliminary study (50 g xylitol in 300 mL) led to bloating and diarrhea in 60–70% of all subjects up to two hours after administration [5]. Other potential side effects associated with sweetener consumption are an increase in uric acid and blood lipid concentrations [6,7], which has been associated with obesity and metabolic syndrome [8]. The few studies that have investigated the effects of xylitol on uric acid [9,10,11,12] and blood lipid [12,13,14] concentrations in humans so far show increased concentrations in response to acute ingestion and mixed results after chronic exposure.

The primary objective of the present study was to determine whether a dose-dependent effect in the release of CCK, aGLP-1 and PYY (peptide tyrosine tyrosine) can be observed for xylitol. The secondary objectives were the effects on the secretion of motilin, GIP (glucose-dependent insulinotropic peptide) and glucagon, as well as on gastric emptying rates, in order to get a more complete picture of the effect of xylitol in the release of gut hormones, the control of glycemic control, as well as its satiating potential. Finally, possible side effects such as gastrointestinal symptoms, impact on uric acid and blood lipid profile were also studied.

With this study we show that xylitol exhibits a combination of properties (weak effect on blood sugar levels but significant release of satiation hormones) that render this sweetener an attractive sugar substitute.

## 2. Materials and Methods

### 2.1. Study Approval

The protocol was approved by the Local Ethical Committee of Basel (Ethikkommission Nordwest- und Zentralschweiz (EKNZ): 2016-01928). The study was performed in accordance with the principles of the Helsinki Declaration of 1975 as revised in 1983. Each subject gave written informed consent for the study. The study was registered at ClinicalTrials.gov (NCT03039478).

### 2.2. Subjects

A total of 12 normal weight, healthy participants (mean body-mass-index (BMI): 21.2 ± 0.4 kg/m^2^, range 19.4–23.0 kg/m^2^, 5 men and 7 female; mean age: 26.4 ± 1.5 years, range 24–42 years) participated in the study. See participant flowchart (Appendix A: flow diagram). Exclusion criteria included substance and alcohol abuse, regular intake of medications (except oral contraceptives), acute infections, chronic medical illness or illnesses affecting the gastrointestinal system. None of the subjects had a history of food allergies, dietary restrictions or pre-existing consumption of xylitol on a regular basis. Weight, height and BMI were recorded for all participants.

### 2.3. Study Design and Experimental Procedure

This study was conducted as a randomized (counterbalanced), placebo-controlled, double-blind, cross-over trial. On four separate occasions, at least 3 days apart and after a 10 h overnight fast, participants were admitted to the St. Clara Research Ltd., at ~0800 AM. A feeding tube was placed transnasally, the intragastric position was confirmed by rapid injection of 10 mL of air and auscultation of upper abdomen. The rationale for intragastric administration of the nutrients was to bypass exteroceptive cues (e.g., taste and smell) and associated hedonic responses and cognitions that may influence subjective ratings or even physiological/endocrine responses [15]. An antecubital catheter was inserted into a forearm vein for blood collection. The subjects were positioned in a comfortable sitting position with their knees bent (80°) and trunk upright.

After taking fasting blood (t = −10 and −1 min) and breath samples (t = −10), as well as assessing appetite-related perceptions and GI symptoms before treatment, participants received one of the following test solutions (at t = 0 min) directly into the stomach over 2 min in randomized order:

- 7 g xylitol dissolved in 300 mL tap water
- 17 g xylitol dissolved in 300 mL tap water
- 35 g xylitol dissolved in 300 mL tap water
- 300 mL tap water (placebo)

For the determination of the gastric emptying rates, 50 mg of ^13^C-sodium acetate was added to the different test solutions. The intragastric infusions were freshly prepared each morning of the study and were at room temperature when administered. The study participant and the person who carried out all tests, as well as the personnel performing analysis of blood samples, were blinded concerning the content of the intragastric infusion administered.

After the administration of the test solutions, blood samples (at t = 15, 30, 45, 60, 90, 120, and 180 min; for analysis of plasma CCK, aGLP-1, PYY, GIP, motilin, glucose, insulin, and glucagon) and breath samples (at t = 15, 30, 45, 60, 75, 90, 105, 120, 150, 180, 210, and 240 min; for analysis of gastric emptying rates) were taken. Extra blood samples were taken during the visit with the highest xylitol load (35 g) at t = 30, 60, and 90 min for analysis of serum total cholesterol, high- (HDL) and low-density lipoprotein (LDL), triglyceride and uric acid concentrations.

Appetite-related sensations (hunger, prospective food consumption, satiety and fullness) were scored immediately after each blood collection by use of previously validated visual analog scales (VASs) [16,17], which consist of a horizontal, unstructured, 10 cm line with words anchored at each end, expressing the most positive and most negative rating (e.g., for hunger: 0 cm: not at all hungry, 10 cm: as hungry as I have ever felt).

Participants were also asked to rate gastrointestinal symptoms at t = 30, 60, 90, 120, 150, 180, and 240 min after the administration of the test solutions. Gastrointestinal symptoms were assessed by the use of a checklist including the following questions: (a) abdominal pain, (b) nausea, (c) vomiting, (d) diarrhea, (e) borborygmus, (f) abdominal bloating, (g) eruction and (h) flatulence. Participants were asked to choose between “no symptom” (0 points), “mild symptoms” (1 point) and “severe symptoms” (2 points) for each question at each time point.

### 2.4. Materials

Xylitol was purchased from Mithana GmbH (Switzerland) and ^13^C-sodium acetate from ReseaChem (Burgdorf, Switzerland).

### 2.5. Blood Sample Collection and Processing

CCK, aGLP-1, PYY, and GIP blood samples were collected on ice into tubes containing EDTA (6 µmol/L blood), a protease-inhibitor cocktail (Complete, EDTA-free, 1 tablet/50 mL blood, Roche, Mannheim, Germany), and a dipeptidyl peptidase IV inhibitor (10 µL/mL blood, Millipore Corp., St. Charles, Missouri, USA). Insulin, glucose, and glucagon blood samples were collected on ice into tubes containing EDTA (6 µmol/L blood) and a protease-inhibitor cocktail (Complete, EDTA-free, 1 tablet/50 mL blood, Roche, Mannheim, Germany). Motilin blood samples were collected on ice into tubes containing lithium heparine and a protease-inhibitor cocktail (Complete, EDTA-free, 1 tablet/50 mL blood, Roche, Mannheim, Germany). Blood lipids and uric acid blood samples were collected on ice into serum tubes. After centrifugation (4 °C at 3000 rpm for 10 min), plasma samples were processed into different aliquots and serum samples left standing for 30 min before they were processed into different aliquots as well. All samples were stored at −80 °C until analysis.

### 2.6. Assessment of Gastric Emptying Rates

The gastric emptying rate was determined using a ^13^C-sodium acetate breath test, an accurate, non-invasive method for measuring gastric emptying, without radiation exposure, and a reliable alternative to scintigraphy, the current “gold standard” [18]. Test solutions were labeled with 50 mg of ^13^C-sodium acetate, an isotope absorbed readily in the proximal small intestine, next transported to the liver where it is metabolized to ^13^CO_2_, which is then exhaled rapidly [18]. At fixed time intervals, end-expiratory breath samples were taken into a 100 mL foil bag. The ^13^C-exhalation was determined by non-dispersive infrared spectroscopy usin g an isotope ratio mass spectrophotometer (Kibion^®^ Dynamic Pro; Kibion GmbH, Bremen, Germany), and expressed as the relative difference (δ ‰) from the universal reference standard (carbon from Pee Dee Belemnite limestone). ^13^C-enrichment was defined as the difference between pre-prandial ^13^C-exhalation and post-prandial ^13^C-exhalation at defined time points, δ over basal (DOB, ‰). Delta values were converted into atom percent excess and then into percent of administered dose of ^13^C excreted per hour (%dose/h (%)). Kinetics emptying was assessed by non-linear regression analysis using the Origin 2019 Software (OriginLab Corp., Northampton, MA, USA) and the modified Ghoos equation [18]:

(1) $$y\left(t\right)=m\cdot k\cdot \beta \cdot \exp\left(-k\cdot \left(t-t_{0}\right)\right)\cdot {\left(1-\exp\left(-k\cdot \left(t-t_{0}\right)\right)\right)}^{\left(\beta -1\right)}$$

(2) $$T_{10}=t_{0}+\left(-1\right)/k\cdot \ln\left(1-0.1^{\left(1/\beta \right)}\right)$$

(3) $$T_{50}=t_{0}+\left(-1\right)/k\cdot \ln\left(1-0.5^{\left(1/\beta \right)}\right)$$

where *k* and β are parameter of the curve, m denotes the extent of marker emptied, T10 and T50 denotes the times, were 10 and 50% of the marker is emptied, and t0 the lag-time of gastric emptying. For goodness of fits, coefficient of determination, R^2^ are reported.

### 2.7. Laboratory Analysis

Plasma CCK: was measured with a sensitive radioimmunoassay using a highly specific antiserum (No. 92128) [19]. The intra- and inter-assay variability is below 15%, respectively.

Plasma active GLP-1: was quantified using a non-radioactive high-sensitive sandwich ELISA (Millipore EZGLPHS-35K) in the presence of a chemiluminescent substrate. The intra- and inter-assay variability is below 9% and 13%, respectively.

Plasma PYY-3-36 and GIP: were measured with sandwich ELISA kits from Millipore (Human GIP (total)-# EZHGIP-54K, Human PYY-3-36 (total)-# EZHPYYT66K). The intra- and inter-assay variability for PYY is below 5.78% and 16.50%, respectively. The intra- and inter-assay variability for GIP is below 8.8% and 6.1%, respectively.

Plasma motilin: was measured as previously described using 125I (Nle13) human motilin as tracer and rabbit anti-human Nle13 motilin antibody (final dilution 1/100000) [20]. The intra- and inter-assay variability is below 4.9% and 5.9%, respectively.

Plasma glucose: was measured by a glucose oxidase method (Rothen Medizinische Laboratorien AG, Basel, Switzerland).

Plasma insulin: was quantified using a chemiluminescent microparticle immunoassay (chemiflex) reagent kit (#8k41; Abbott) and the relative light units detected by the ARCHITECT optical system (model: CI4100; Abbott). The ARCHITECT Insulin assay precision is ≤7% total CV.

Plasma glucagon: was measured after extraction of plasma with 70% ethanol (vol/vol, final concentration). The antibody is directed against the C-terminus of the glucagon molecule (antibody code no. 4305) and therefore mainly measures glucagon of pancreatic origin [21,22]. The intra- and inter-assay variability is below 6% and 15%, respectively.

Serum blood lipids (serum triglyceride, cholesterol, low-density lipoprotein (LDL) and high-density lipoprotein (HDL)): were measured with enzymatic assays from Beckman-Coulter (Rothen Medizinische Laboratorien AG, Basel, Switzerland). The intra and inter-assay variability is below 1.1% and 1.2% (triglyceride), below 0.8% and 1.1% (cholesterol), below 0.9% and 0.9% (LDL), and below 1.0% and 1.8% (HDL).

Serum uric acid: was measured with an enzymatic assay from Beckman-Coulter (Rothen Medizinische Laboratorien AG, Basel, Switzerland). The intra- and inter-assay variability is below 0.7% and 0.8%.

### 2.8. Statistics

In our previous work investigating the effects of 50 g xylitol on gut hormone secretion and gastric emptying rates, we did find significant results [5]. However, since pharmacological effects are usually not linear with respect to dose, we did not have a reliable estimate of effect size for the current study, so no formal estimate of sample size could be obtained. A sample size of 12 subjects per group was chosen for reasons of comparability and practicability. In this respect, this study is a hypothesis-generating study that allows descriptive data analysis.

Descriptive statistics were used for demographic variables such as age, weight, height and BMI. For hormone and glucose profiles, as well as gastric emptying rates and appetite-related sensations, incremental values were used to calculate area under the curve (iAUC) by trapezoidal rule. Maximum and minimum deviations from baseline-iCmax and iCmin—were determined on baseline-corrected data, as well. For AUC calculations, in addition to the total time interval of 180 min, an interval of 60 min is reported as in some parameters (CCK, aGLP-1) the main effect is seen in this time period. Linear mixed-effects modeling was applied to describe differences between the different treatments (placebo, 7, 17 and 35 g xylitol). In case of significant overall treatment effects, pairwise post-hoc within-subject comparisons were done using Šidak multicomparison test. In addition, for the parameters of interest (e.g., the iAUCs 0–60 min for CCK, aGLP-1 and PYY) the minimum detectable differences were estimated on the basis of the observed data in the present study by a simulation using the PASS 2020 Power Analysis and Sample Size Software (2020), NCSS, LLC, Kaysville, UT, USA (using 1000 iterations per each run).

To explore putative relationships between different gut hormone responses (e.g., CCK, aGLP-1, and PYY) and gastric emptying of the different treatments, the integrated responses (iAUC 0–60 min) were correlated on an individual basis by linear matrix correlation. The goodness of this correlation was expressed by Pearson’s correlation coefficient, R.

All statistical analysis was done using the statistical software package IBM SPSS Statistics for Windows, Version 25.0. Armonk, NY: IBM Corp. Values were reported as means ± standard deviation (SD), and displayed in figures as means ± standard error of mean (SEM). Differences were considered to be statistically significant when *p* < 0.05.

## 3. Results

All subjects tolerated the study well and there were no adverse events that would have led to study discontinuation. Complete data from 12 participants were available for analysis.

### 3.1. Fasting Values

For each measured parameter, fasting values were compared between the treatments. None of these parameters were statistically significant between the treatments.

### 3.2. Gut Hormone Secretion

**Figure 1:**
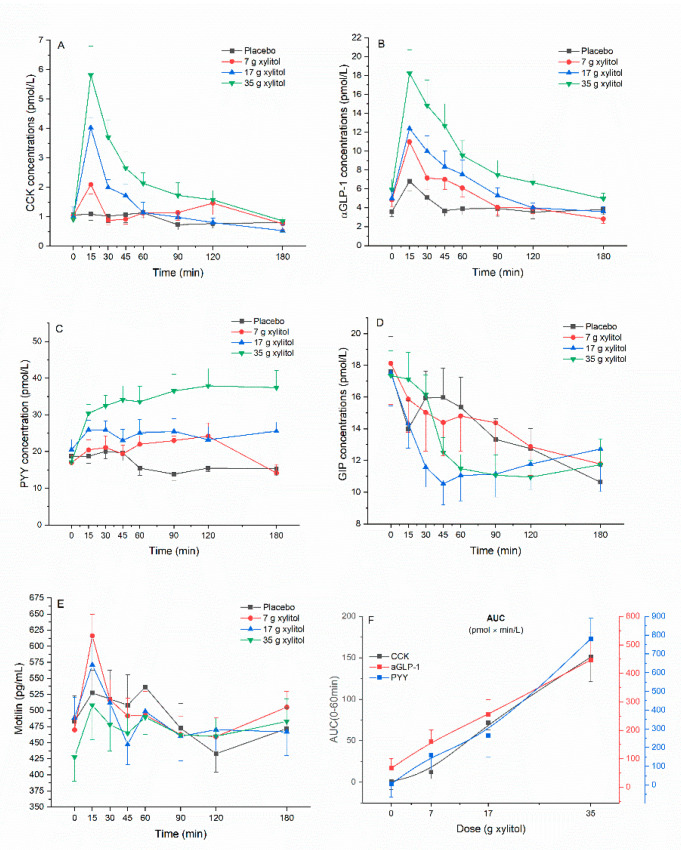
(**A**) cholecystokinin (CCK), (**B**) active glucagon-like peptide-1 (aGLP-1), (**C**) peptide tyrosine tyrosine (PYY), (**D**) glucose-dependent insulinotropic peptide (GIP), (**E**) motilin, and (**F**) dose–response evaluation. Data are expressed as mean ± SEM, absolute values are reported. N = 12 (5 men and 7 women). Statistical tests: Linear mixed-effects modeling followed by Šidak post-hoc test in case of overall significance. Results of the statistical analysis are shown in Table 1.

Plasma CCK: Xylitol leads to a dose-dependent increase in plasma CCK concentrations compared to placebo. There was an overall statistically significant difference comparing iAUC 0–60 min (*p* < 0.001). Pairwise comparison revealed statistically significant difference for placebo vs. 17 g xylitol (*p* < 0.001), placebo vs. 35 g xylitol (*p* = 0.002), 7 vs. 17 g xylitol (*p* = 0.009), and 7 vs. 35 g xylitol (*p* = 0.010). Overall statistical significance was also reached for iAUC 0–180 min (*p* = 0.026). Pairwise comparisons revealed statistically significant difference for placebo vs. 35 g xylitol (*p* = 0.023). Further, there was an overall statistically significant difference comparing iCmax (*p* < 0.001): pairwise significant differences were observed between placebo vs. 17 g xylitol (*p* < 0.001), placebo vs. 35 g xylitol (*p* = 0.002), 7 vs. 17 g xylitol (*p* = 0.009), and 7 vs. 35 g xylitol (*p* = 0.010) (Figure 1A, Table 1).

Plasma aGLP-1: Xylitol leads to a dose-dependent increase in plasma aGLP-1 concentrations compared to placebo. There was an overall statistically significant difference comparing iAUC 0–60 min (*p* = 0.002). Pairwise comparison revealed statistically significant difference for placebo vs. 17 g xylitol (*p* = 0.008), and placebo vs. 35 g xylitol (*p* = 0.006). Further, there was an overall statistically significant difference comparing iCmax (*p* = 0.002): pairwise significant differences were observed between placebo vs. 17 g xylitol (*p* = 0.013), and placebo vs. 35 g xylitol (*p* = 0.003) (Figure 1B, Table 1).

Plasma PYY: Xylitol leads to a dose-dependent increase in plasma PYY concentrations compared to placebo. There was an overall statistically significant difference comparing iAUC 0–60 min (*p* < 0.001). Pairwise comparison revealed statistically significant difference for placebo vs. 35 g xylitol (*p* = 0.002), 7 vs. 35 g xylitol (*p* < 0.001), and 17 vs. 35 g xylitol (*p* = 0.005). Overall statistical significance was also reached for iAUC 0–180 min (*p* = 0.004). Pairwise comparisons revealed statistically significant difference for placebo vs. 35 g xylitol (*p* = 0.003), 7 vs 35 g xylitol (*p* = 0.015), and 17 vs. 35 g xylitol (*p* = 0.007). Further, there was an overall statistically significant difference comparing iCmax (*p* = 0.001): pairwise significant differences were observed between placebo vs. 7 g xylitol (*p* = 0.030), placebo vs. 35 g xylitol (*p* = 0.002), and 17 vs. 35 g xylitol (*p* = 0.024). (Figure 1C, Table 1).

Plasma GIP: No effect of xylitol treatment on plasma GIP concentrations could be found: the values of iAUC 0–60 min, iAUC 0–180 min and iCmin did not show any overall statistically significant differences (Figure 1D, Table 1).

Plasma motilin: No effect of xlylitol treatment on plasma motilin concentrations could be found: the values of iAUC 0–60 min, iAUC 0–180 min and iCmin did not show any overall statistically significant differences (Figure 1E, Table 1).

### 3.3. Minimum Detectable Difference

Based on the results of this study, the minimum detectable differences were estimated for the incremental AUCs 0–60 min for CKK, aGLP-1 and PYY. The minimum detectable difference for CCK was 68.0 pmol × min/L, for aGLP-1 162 pmol × min/L, and finally for PYY 332 pmol × min/L with a corresponding statistical power (95% confidence interval) of 80.7% (78.1–83.1%), 80.9 % (78.3–83.3%) and 82.3% (79.8–84.6%), respectively. These data correspond well with the applied statistical tests.

### 3.4. Dose–Response Evaluation

For CCK, aGLP-1 and PYY the dose–response relationship was evaluated and shown in Figure 1F. On average, all these hormones showed a monotonically increasing curvilinear secretion with increasing xylitol dose. For none of the hormones an apparent saturation of the stimulating effect was observed, so that no half-maximal stimulating dose (ED50) could be estimated.

### 3.5. Gastric Emptying

**Figure 2:**
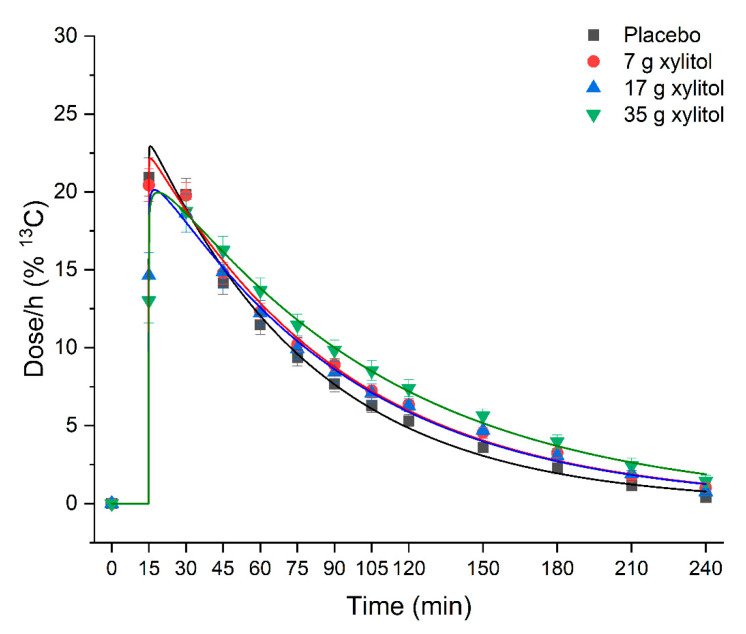
Effect of xylitol on gastric emptying. Data are expressed as mean ± SEM. N=12 (5 men and 7 women). Kinetic data was analyzed by non-linear regression analysis using a modified Ghoose model for gastric emptying [18]. Statistical tests: Linear mixed-effects modeling followed by Šidak post-hoc test in case of overall significance. Goodness of fit is indicated by the coefficient of determination R^2^: placebo R^2^ = 0.992, 7 g xylitol R^2^ = 0.995, 17 g xylitol R^2^ = 0.995, 35 g xylitol R^2^ = 0.999. Results of the statistical analysis are shown in Table 2.

Xylitol leads to a dose-dependent deceleration of gastric emptying rates compared to placebo (Figure 2, Table 2). There was an overall statistically significant difference comparing T50% (*p* = 0.026). Pairwise comparisons revealed that time needed to empty 50% of the dose was significantly shorter for placebo vs. 35 g xylitol (56.6 ± 13.0 min vs. 74.0 ± 18.1 min; *p* = 0.029). Goodness of fit parameter R2 were between 0.992 and 0.999 for all treatments.

### 3.6. Correlations Gastric Emptyimg Rates and Gut Hormone Concentrations

**Figure 3:**
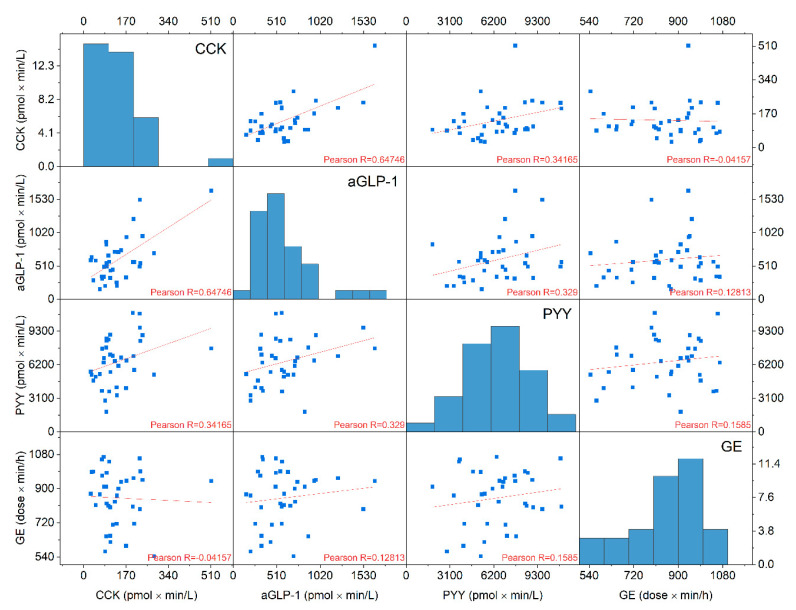
Correlation matrix of cholecystokinin (CCK), active glucagon-like peptide-1 (aGLP-1), peptide tyrosine tyrosine (PYY), and gastric emptying (GE). iAUC 0–60 min values from n = 36 samples from all xylitol treatments were used. CCK, aGLP-1 and PYY were weakly negatively correlated to gastric emptying (Pearson’s R=−0.041, R = 0.128 and R = 0.158, respectively). In the diagonal, histograms of distribution of individual values are shown. The x-axes of the histograms are identical with that of other plots of the same column. The y-axes of the histogram denotes the number of individual samples iAUC values in the interval of the width of the respective histogram bar.

CCK, aGLP-1 and PYY were weakly linear correlated to gastric emptying (R = −0.041, R = 0.128 and R = 0.158, respectively) (Figure 3).

### 3.7. Appetite-Realted Sensations

**Figure 4:**
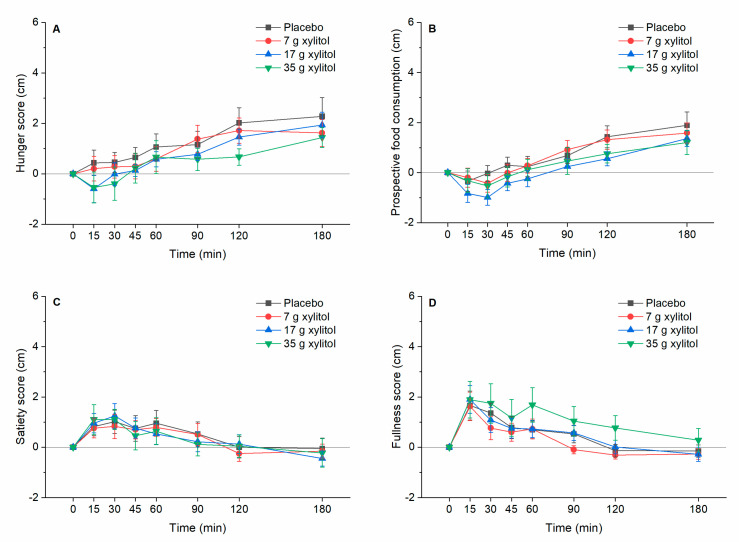
(**A**) hunger, (**B**) prospective food consumption, (**C**) satiety, and (**D**) fullness. Data are expressed as mean ± SEM, baseline values are reported. N = 12 (5 men and 7 women). Statistical tests: Linear mixed-effects modeling followed by Šidak post-hoc test in case of overall significance.

Appetite-related sensations: No effect of xylitol treatment on appetite-related sensations could be found: The values of iAUC 0–60 min, iAUC 0–180 min and iCmax did not show any overall statistically significant differences (Figure 4A–D).

### 3.8. Plasma Glucose, Insulin and Glucagon Release

**Figure 5:**
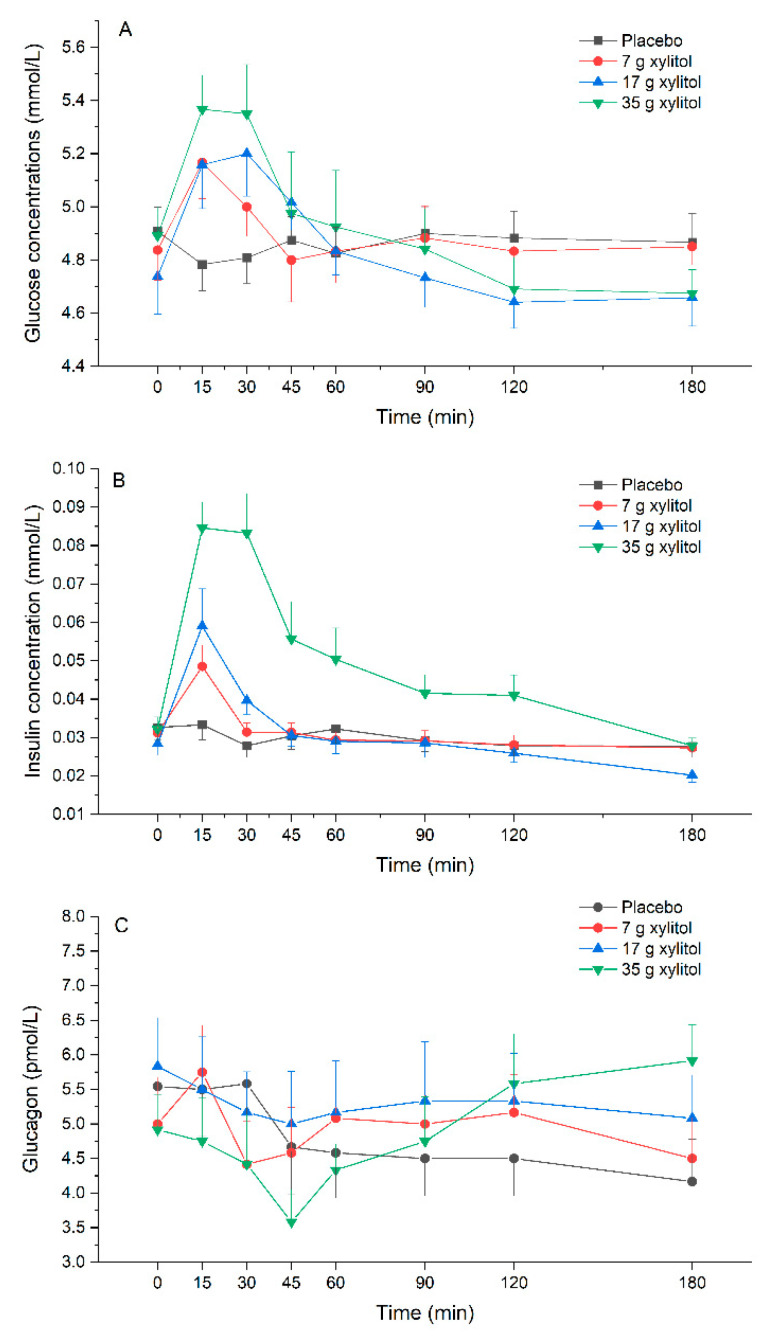
(**A**) glucose, (**B**) insulin, and (**C**) glucagon. Data are expressed as mean ± SEM, absolute values are reported. N=12 (5 men and 7 women). Statistical tests: Linear mixed-effects modeling followed by Šidak post-hoc test in case of overall significance. Results of the statistical analysis are shown in Table 3.

Plasma glucose: Xylitol leads to a dose-dependent increase in plasma glucose concentrations compared to placebo. There was an overall statistically significant difference comparing iAUC 0–60 min (*p* < 0.001). Pairwise comparison revealed statistically significant difference for placebo vs. 17 g xylitol (*p* < 0.001). Further, there was an overall statistically significant difference comparing iCmax (*p* < 0.001): pairwise significant differences were observed between placebo vs. 17 g xylitol (*p* < 0.001), and placebo vs. 35 g xylitol (*p* = 0.001) (Figure 5A, Table 3).

Plasma insulin: Xylitol leads to a dose-dependent increase in plasma insulin concentrations compared to placebo. There was an overall statistically significant difference comparing iAUC 0–60 min (*p* < 0.001). Pairwise comparison revealed statistically significant difference for placebo vs. 17 g xylitol (*p* < 0.001), placebo vs. 35 g xylitol (*p* < 0.001), 7 vs. 35 g xylitol (*p* = 0.001), and 17 vs. 35 g xylitol (*p* = 0.004). Overall statistical significance was also reached for iAUC 0–180 min (*p* = 0.008). Pairwise comparisons revealed statistically significant difference for placebo vs. 35 g xylitol (*p* = 0.010), 7 vs. 35 g xylitol (*p* = 0.006), and 17 vs. 35 g xylitol (*p* = 0.008). Further, there was an overall statistically significant difference comparing iCmax (*p* < 0.001): pairwise significant differences were observed between placebo vs. 17 g xylitol (*p* = 0.030), placebo vs. 35 g xylitol (*p* < 0.001), 7 vs. 35 g xylitol (*p* = 0.001), and 17 vs. 35 g xylitol (*p* = 0.036) (Figure 5B, Table 3).

Plasma glucagon: There was an overall statistically significant difference comparing iCmax (*p* = 0.011). Pairwise comparison revealed statistically significant difference for 17 vs. 35 g xylitol (*p* = 0.012).

### 3.9. Impact on Blood Lipid Profil, Uric Acid and Gastrointestinal Symptoms

Blood lipids: There was no statistically significant increase from baseline in blood lipids (total cholesterol, LDL, HDL and triglycerides) within 120 min after 35 g xylitol. Mean fasting concentration of total cholesterol was 4.2 ± 0.2 mmol/L (Cmax 4.4 ± 0.2 mmol/L), LDL 2.5 ± 0.2 mmol/L (Cmax 2.5 ± 0.2 mmol/L), HDL 1.4 ± 0.1 mmol/L (Cmax 1.5 ± 0.1 mmol/L), and triglycerides 0.9±0.1 mmol/L (Cmax 0.9±0.1 mmol/L) (data not shown).

**Figure 6:**
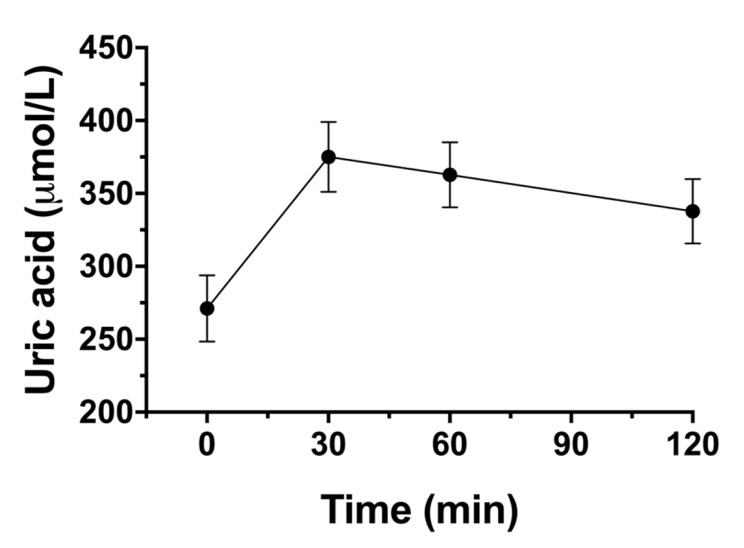
Effect of the highest xylitol load (35 g) on uric acid concentrations. Data are expressed as mean ± SEM, absolute values are reported. N = 12 (5 men and 7 women). Statistical tests: Linear mixed-effects modeling followed by Šidak post-hoc test in case of overall significance.

Uric acid: There was a statistically significant increase from baseline in uric acid concentrations after 35 g xylitol (*p* < 0.001). Mean fasting concentration uric acid was 271.1 ± 22.8 µmol/L (Cmax 376.2 ± 23.6 mmol/L) (Figure 6).

Gastrointestinal symptoms: There was no abdominal pain, nausea, vomiting, diarrhea, or flatulence after any dose and no difference between xylitol and placebo was found. In five participants after placebo, nine participants after 7 g, eight participants after 17 g and nine participants after 35 g xylitol a subjective increase in bowel sounds was reported (max. reported severity 1.4 after placebo), and two volunteers described an increased feeling of bloating after 17 g and 35 g xylitol (maximum reported severity 1.0). One volunteer had eructation after 7 g (reported severity 1.0) (Table 4).

## 4. Discussion

The main results of the study can be summarized as follows: (i) xylitol induces a dose-dependent stimulation of CCK, aGLP-1, and PYY release, and deceleration of gastric emptying rates, (ii) xylitol leads to a dose-dependent increase in blood glucose and insulin concentrations, (iii) xylitol has no effect on motilin, glucagon or GIP-release, (iv) blood lipids are not affected by 35 g xylitol, but a rise in uric acid is observed, and (v) doses up to 35 g xylitol are well tolerated.

The gastrointestinal tract plays an important role in regulating appetite and satiation and thus maintaining energy balance. Nutrient components such as carbohydrates, fats and amino acids are recognized by receptors located on enteroendocrine cells in the intestinal tract, which stimulate the release of gut hormones such as CCK, GLP-1, PYY, and GIP. These hormones are able to activate a whole range of satiation mechanisms, including deceleration of gastric emptying [23,24].

Xylitol provides around half the calories of sucrose per gram. However, most of the substance ingested is never absorbed and the bioavailability is therefore significantly lower. Nevertheless, in our previous trial with 50 g xylitol we could show for the first time, that the satiation cascade is stimulated: although low in calories, xylitol leads to a strong increase in the gut hormones CCK and GLP-1, and subsequently delays gastric emptying [5]. Other low- or non-caloric sweeteners (e.g., aspartame, acesulfame-K and sucralose) have no stimulating effect on gut hormone release in vivo [25,26,27]. With the present study we substantiate that xylitol induces a rise in plasma gut hormones and delays gastric emptying and expand these findings by showing a stimulation of CCK, aGLP-1 and PYY in a dose-dependent manner. Gastric emptying is regulated by numerous feedback mechanisms, including gut hormone release such as CCK and GLP-1 [23,24]. In this trial, xylitol induced a deceleration of gastric emptying which is at least partly mediated via the gut hormones as we were able to show that CCK, aGLP-1 and PYY correlated to gastric emptying. The underlying mechanism of the xylitol induced gut hormone secretion is not clear yet, however, we infer from these observations that the ability of sweeteners to induce a secretion of gut hormones is sweetener-specific, irrespective of its caloric value. A recent publication indicates that increase in duodenal osmolarity by administration of a hyperosmolar saline solution is also associated with an increase in CCK, GLP-1 and PYY concentrations, while GIP and glucagon are not affected [28]. Whether osmolarity plays a role in the xylitol-stimulated gut hormone release is not known at this point. In our trial, the solution with the lowest concentration (7 g) was hypoosmolar (153.34 mOsmol/L), yet clearly stimulated CCK and GLP-1 release. We therefore assume that osmolarity alone is not responsible for the xylitol-stimulated release of gut hormones.

In our trial, we have also investigated the effect of xylitol on plasma motilin concentrations, another gut hormone involved in the control of appetite and food intake. Plasma motilin concentrations fluctuate in synchrony with the phases of the migrating motor complex, and reach a peak just before the occurrence of a gastric phase III [29,30,31]. Motilin-induced gastric phase III contractions signal hunger and plasma motilin concentrations are closely associated with interdigestive hunger ratings [32]. In a recent study we investigated the effect of two caloric sweeteners—glucose and fructose—versus the non-caloric sweetener acesulfam-K on plasma motilin concentrations and showed that glucose and fructose inhibited motilin secretion, which can be interpreted as a satiation signal [27]. In contrast, acesulfam-K had no inhibitory effect on motilin. In the present study, the effect of xylitol on motilin concentrations was not different from tap water. This combination of properties—stimulation of gut hormones such as CCK, GLP-1, and PYY, but no inhibition of motilin—means that xylitol occupies an intermediate position between caloric- and non-caloric sweeteners. In 1989, Salminen et al. [4] compared 30 g glucose vs. 30 g xylitol ingestion on gastric emptying, GIP and motilin release. They described an early (at 20 min), transient but significant increase in plasma motilin concentrations after xylitol intake. However, in our current trial as well as in the study carried out by Salminen et al., no gastroduodenal manometry was performed, which would have allowed to adjust motilin measurements around a phase III of the MMC. Therefore, the question whether xylitol has an inhibitory or a stimulating effect on motilin release is still not clear and needs to be addressed in further studies.

An emerging number of studies indicate that sweeteners—whether caloric or non-caloric—can influence glycemic control both positively and negatively. Xylitol was found to exert anti-hyperglycemic effects when given over a longer period of time in diabetic rats, possibly via enhancing insulin-mediated glucose uptake and reducing intestinal glucose absorption [33]. Additionally, trophic effects on pancreatic islet cells and strong anti-oxidative potential against T2D-associated oxidative stress could be shown in rodent models [34,35]. Human studies measuring glucose levels in patients with diabetes before and after regular administration of xylitol over a long period of time are not currently available. In our study, after acute administration of xylitol in healthy volunteers only a weak increase in blood glucose levels was observed.

Another important player in glycemic control is the pancreatic hormone glucagon—a hormone produced in the α-cells of the pancreatic islets—which stimulates hepatic glycogenolysis and gluconeogenesis in hypoglycemic states to restore glucose homeostasis, and in this way counter-balances insulin. Effects of acute or chronic intake of xylitol on glucagon secretion have not been studied so far. In the current trial, we found no statistically significant effects of different doses of xylitol on plasma glucagon concentrations in this group of lean, healthy volunteers. However, the highest xylitol dose (35 g) lead to numerically and statistically significant higher late (120 min) glucagon concentrations compared to 17 g without a concomitant increase in plasma glucose concentrations. In order to be able to make a definitive statement as to whether or not xylitol has any effect on glucagon release, further studies would certainly be useful, preferably with longer observation times (>180 min).

Gastrointestinal tolerance was not the main focus of this trial. However, as in literature gastrointestinal side-effects are often mentioned, we recorded them in detail. In our previous trial, a rather high dose of xylitol (50 g) was used, which lead to gastrointestinal side effects such as diarrhea in about 70% of the participants [5]. Previous studies with volunteers unaccustomed to xylitol intake, recommend doses up to maximum 0.3 g/kg body weight [36], which is slightly less than the highest dose (35 g) we administered in the current trial. Moreover, the dose was rapidly applied (over 2 min) in a solution directly into the stomach, which probably causes the greatest possible strain on the gastrointestinal system. In spite of this, 35 g xylitol in 300 mL (the sweetness of this solution corresponds to around 30 g sucrose in 33cl, as found in sweet beverages) was very well tolerated, with subjective increases in bowel sounds as the most common finding (while there was no abdominal pain, vomiting, nausea, diarrhea, or flatulence). Other potential side effects associated with sweetener consumption are alterations in uric acid and blood lipid concentrations. Uric acid has been associated with obesity and metabolic syndrome as well as chronic kidney disease and arterial hypertension [8]. Elevation of uric acid after oral intake of 50 g xylitol has been described by Forster et al. in 1971 [9]. Additionally, intravenous administration of xylitol solutions resulted in an increase in plasma uric acid [10]. In chronic exposition trials with insulin-dependent children receiving 30 g xylitol/day an increase in uric acid was found, however in healthy adult volunteers, 80–100 g of xylitol/day for 18 days had no effect on uric acid, and in the Turku studies with chronic xylitol exposition over 2 years, no effect on uric acid was found either [11,12]. In our trial, we found a 36% increase in uric acid concentrations within the first 30 min which did not return to baseline values until the last blood sample was taken after 120 min. Although mean serum uric acid levels were within the normal range at all time points, the observed increase in uric acid has to be investigated further, especially when consumed on a regular basis, and patients with gout should probably be careful. At least in theory, also triglyceride synthesis might be affected by ingestion of xylitol: The glycerol backbone-which is found in triglycerides-is a product of the pentose-phosphate pathway. In this way, glycerol concentrations might increase which might end up in triglyceride synthesis. Only a few studies have examined the effects of xylitol intake on blood lipids in humans so far. Acute effects of enteral administration of a xylitol containing formula in lean volunteers lead to an increase in triglycerides and very low-density lipoproteins [13]. In trials with chronic exposition, no effect on blood lipid concentrations could be found [12,14]. In the current trial, we examined blood lipid profile after ingestion of the highest dose—35 g xylitol—and did not find any changes from baseline values.

Some potential limitations need to be addressed: In this trial, we studied acute effects of single bolus doses of xylitol applied in a liquid in normal weight subjects who were not used to these substances. Differential effects of long-term exposure on the secretion of gut hormone and gastric emptying rates need to be investigated, as adaptive processes cannot be ruled out. Furthermore, endogenous motilin secretion fluctuates in synchrony with antral contractility of the migrating motor complex and reaches a peak just before gastric phase III [31]. In this trial, blood samples were not taken in synchrony with phase III, and therefore interpretation of motilin results is difficult and motilin responses ought to be studied further. Finally, while we did not find any impact on blood lipids, and only mild gastrointestinal symptoms even when applying the highest dose of xylitol in normal weight healthy volunteers, future trials should probably include the target audience (obese patients with metabolic syndrome) studied over a longer period of time (chronic intake) with special attention to uric acid.

## 5. Conclusions

The present study shows that even low doses (7 to 35 g) of the natural sweetener xylitol dose-dependently stimulate the secretion of CCK, aGLP-1 and PYY, and decelerate gastric emptying rates in lean participants. Meanwhile, there is only little effect on plasma glucose and insulin, and no effect on motilin, GIP, or blood lipid concentrations. Circulating uric acid concentrations increase acutely following xylitol consumption, the clinical implications are unclear and further research is necessary on this topic. Given as a single bolus dose in a liquid, doses up to 35 g xylitol were well tolerated in this trial. With this study we show that xylitol exhibits a combination of properties (weak effect on blood sugar levels but significant release of satiation hormones) that render this sweetener an attractive sugar substitute.

## Figures and Tables

**Table 1:** Effect of xylitol on plasma concentrations of CCK, aGLP-1, PYY, GIP and motilin.

Parameters		A: Placebo (n = 12)	B: Xylitol 7 g (n = 12)	C: Xylitol 17 g (n = 12)	D: Xylitol 35 g (n = 12)	*p*-Values (Overall)	*p*-Values (post-hoc)
**CCK**	Fasting values (pmol/L)	1.1 ± 0.8	1.0 ± 0.6	1.0 ± 1.1	0.9 ± 0.7	NS	
	iAUC (0–60 min) (pmol × min/L)	0.6 ± 32.6	12.1 ± 27.6	71.8 ± 30.2	150.8 ± 102.8	*p* < 0.001	A vs. C: *p* < 0.001 A vs. D: *p* = 0.002 B vs. C: *p* = 0.009 B vs. D: *p* = 0.010
	iAUC (0–180 min) (pmol × min/L)	−29.2 ± 108.9	27.1 ± 112.4	49.7 ± 89.4	222.2 ± 187.6	*p* = 0.026	A vs. D: *p* = 0.023
	iCmax (pmol/L)	0.6 ± 0.6	1.4 ± 1.1	3.0 ± 1.2	5.1 ± 3.1	*p* < 0.001	A vs. C: *p* < 0.001 A vs. D: *p* = 0.002 B vs. C: *p* = 0.009 B vs. D: *p* = 0.010
	Tmax (min)	40.0 ± 52.1	32.5 ± 41.4	15.0 ± 0.0	25.0 ± 30.2	NS	
	iCmin (pmol/L)	−0.7 ± 0.6	−0.5 ± 0.4	−0.6 ± 0.7	−0.2 ± 0.4	NS	
	Tmin (min)	90.0 ± 56.1	113.8 ± 74.4	127.5± 62.8	80.0 ± 86.1	NS	
**aGLP**	Fasting values (pmol/L)	3.6 ± 1.9	4.9 ± 2.5	5.0 ± 3.4	5.9 ± 3.8	NS	
	iAUC (0–60 min) (pmol × min/L)	66.5 ± 119.1	160.6 ± 170.2	254.7 ± 182.2	447.0 ± 332.1	*p* = 0.002	A vs. C: *p* = 0.008 A vs. D: *p* = 0.006
	iAUC (0–180 min) (pmol × min/L)	71.8 ± 269.4	53.6 ± 392.4	215.1 ± 330.4	553.5 ± 612.8	NS	
	iCmax (pmol/L)	3.5 ± 2.8	6.9 ± 5.4	8.2 ± 4.2	13.0 ± 7.3	*p* = 0.002	A vs. C: *p* = 0.013 A vs. D: *p* = 0.003
	Tmax (min)	31.3 ± 49.3	28.8 ± 30.3	25.0 ± 14.8	28.8 ± 30.3	NS	
	iCmin (pmol/L)	−1.4 ± 1.0	−2,5 ± 2.5	−2.1 ± 2.5	−1.9 ± 2.6	NS	
	Tmin (min)	98.8 ± 60.2	112.5 ± 69.0	91.3 ± 79.0	105.0 ± 84.4	NS	
**PYY**	Fasting values (pmol/mL)	18.7 ± 6.3	17.0 ± 6.8	20.5 ± 9.2	17.2 ± 6.2	NS	
	iAUC (0–60 min) (pmol × min/L)	5.6 ± 240.0	159.3 ± 241.4	263.1 ± 380.6	780.8 ± 378.6	*p* < 0.001	A vs. D: *p* = 0.002 B vs. D: *p* < 0.001 C vs. D: *p* = 0.005
	iAUC (0–180 min) (pmol × min/L)	−256.5 ± 775.1	537.1 ± 935.6	835.9 ± 1420.4	3148.2 ± 2037.9	*p* = 0.004	A vs. D: *p* = 0.003 B vs. D: *p* < 0.015 C vs. D: *p* = 0.007
	iCmax (pmol/L)	5.1 ± 3.6	13.3 ± 7.4	12.5 ± 8.9	25.7 ± 12.4	*p* = 0.001	A vs. B: *p* = 0.030 A vs. D: *p* = 0.002 C vs. D: *p* = 0.024
	Tmax (min)	30.0 ± 15.7	91.4 ± 47.6	72.3 ± 54.4	88.8 ± 72.8	*p* < 0.001	A vs. B: *p* = 0.009
	iCmin (pmol/L)	−8.4 ± 6.6	−6.9 ± 6.7	−4.4 ± 5.9	−0.3 ± 0.8	*p* = 0.007	A vs. D: *p* = 0.012
	Tmin (min)	115.0 ± 52.5	111.8 ± 82.0	47.7 ± 63.9	30.0 ± 70.1	*p* = 0.030	A vs. D: *p* = 0.047
**GIP**	Fasting values (pmol/L)	17.6 ± 7.67	18.12 ± 8.93	17.51 ± 7.16	17.36 ± 5.44	NS	
	iAUC (0–60 min) (pmol × min/L)	−120.8 ± 295.6	−160.92 ± 234.1	−307.6 ± 194.2	−138.2 ± 191.8	NS	
	iAUC (0–180 min) (pmol × min/L)	−710.9 ± 1186.3	−749.4 ± 925.1	−990.7 ± 815.0	−871.5 ± 727.1	NS	
	iCmax (pmol/L)	3.1 ± 4.6	3.0 ± 3.1	3.2 ± 4.3	3.1 ± 3.5	NS	
	Tmax (min)	37.5 ± 43.6	56.3 ± 58.7	65.0 ± 70.7	30.0 ± 53.5	NS	
	iCmin (pmol/L)	−8.9 ± 6.3	−9.2 ± 6.3	−10.8 ± 5.3	−8.7 ± 4.3	NS	
	Tmin (min)	122.5 ± 62.9	117.5 ± 67.3	102.5 ± 54.6	123.8 ± 46.2	NS	
**Motilin**	Fasting values (pg/mL)	483 ± 136	470 ± 79	489 ± 108	428 ± 136	NS	
	iAUC (0–60 min) (pg × min/mL)	1957 ± 6146	3392 ± 3231	896 ± 4037	2973 ± 4027	NS	
	iAUC (0−180 min) (pg × min/mL)	−182 ± 10963	4091 ± 6991	−1493 ± 11287	8074 ± 11825	NS	
	iCmax (pg/mL)	117.3 ± 116.6	165.6 ± 92.0	119.0 ± 79.2	131.4 ± 75.6	NS	
	Tmax (min)	40.0 ± 23.4	43.8 ± 54.4	36.3 ± 37.5	52.5 ± 53.3	NS	
	iCmin (pg/mL)	−81.9 ± 51.8	−67.8 ± 33.9	−102.3 ± 78.5	−45.0 ± 45.0	NS	
	Tmin (min)	2.3 ± 56.3	21.8 ± 76.9	30.5 ± 91.7	45.4 ± 76.1	NS	

Data are expressed as mean ± SD and reported from baseline (incremental). Statistical tests: Linear mixed-effects modeling followed by Šidak post-hoc test in case of overall significance. aGLP-1, active glucagon-like peptide-1; CCK, cholecystokinin; GIP, glucose-dependent insulinotropic peptide; PYY, peptide tyrosine tyrosine.

**Table 2:** Effect of xylitol on gastric emptying.

	A: Placebo (n = 12)	B: Xylitol 7 g (n = 12)	C: Xylitol 17 g (n = 12)	D: Xylitol 35 g (n = 12)	*p*-Values (Overall)	*p*-Values (post-hoc)
iAUC (0–60 min) (dose × min/h)	911 ± 129	917 ± 101	814 ± 164	823 ± 154	NS	
iAUC (0–180 min) (dose × min/h)	1609 ± 270	1733 ± 221	1614 ± 319	1767 ± 302	NS	
Tmax (min)	21.25 ± 7.7	21.25 ± 7.7	27.50 ± 5.8	30.00 ± 11.1	*p* = 0.039	B vs. C: *p* = 0.099
T50% (time 50% emptied) (min)	56.6 ± 13.0	65.3 ± 11.1	66.9 ± 13.3	74.0 ± 18.1	*p* = 0.026	A vs. D: *p* = 0.029

Data are expressed as mean ± SD and reported from baseline (incremental). Linear mixed-effects modeling followed by Šidak post-hoc test in case of overall.

**Table 3:** Effect of xylitol on plasma concentrations of glucose, insulin and glucagon.

Parameters		A: Placebo (n = 12)	B: Xylitol 7 g (n = 12)	C: Xylitol 17 g (n = 12)	D: Xylitol 35 g (n = 12)	*p*-Values (Overall)	*p*-Values (post-hoc)
**Glucose**	Fasting values (mmol/L)	4.9 ± 0.3	4.8 ± 0.4	4.7 ± 0.5	4.9 ± 0.4	NS	
	iAUC (0–60 min) (mmol × min/L)	−4.5 ± 4.0	6.8 ± 18.6	18.2 ± 0.3	15.5 ± 22.3	*p* < 0.001	A vs. C: *p* < 0.001
	iAUC (0–180 min) (mmol × min/L)	−8.4 ± 14.1	−8.3 ± 56.4	12.8 ± 35.4	−1.0 ± 49.4	NS	
	iCmax (mmol/L)	0.07 ± 0.1	0.37 ± 0.33	0.55 ± 0.21	0.67 ± 0.39	*p* < 0.001	A vs. C: *p* < 0.001 A vs. D: *p* = 0.001
	Tmax (min)	43.8 ± 48.9	31.3 ± 47.6	23.8 ± 10.0	22.5 ± 15.0	NS	
	iCmin (mmol/L)	−0.19 ± 0.07	−0.23 ± 0.25	−0.21 ± 0.16	−0.44 ± 0.31	NS	
	Tmin (min)	55.0 ± 55.9	50.0 ± 54.0	102.5 ± 62.0	97.5 ± 59.8	NS	
**Insulin**	Fasting values (mmol/L)	0.033± 0.012	0.031 ± 0.009	0.028 ± 0.010	0.032 ± 0.011	NS	
	iAUC(0–60 min) (mmol × min/L)	−0.1 ± 0.4	0.3 ± 0.4	0.7 ± 0.4	2.0 ± 1.1	*p* < 0.001	A vs. C: *p* < 0.001 A vs. D: *p* < 0.001 B vs. D: *p* = 0.001 C vs. D: *p* = 0.004
	iAUC (0–180 min) (mmol × min/L)	−0.6 ± 1.2	−0.18 ± 0.3	0.3 ± 0.95	2.9 ± 2.5	*p* = 0.008	A vs. D: *p* = 0.010 B vs. D: *p* = 0.006 C vs. D: *p* = 0.008
	iCmax (mmol/L)	0.01 ± 0.01	0.02 ± 0.01	0.03 ± 0.03	0.07 ± 0.02	*p* < 0.001	A vs. C: *p* = 0.030 A vs. D: *p* < 0.001 B vs. D: *p* = 0.001 C vs. D: *p* = 0.036
	Tmax (min)	41.3 ± 66.8	20.0 ± 22.5	15.0 ± 0.0	25.0 ± 14.8	NS	
	iCmin (mmol/L)	−0.01 ± 0.01	−0.01 ± 0.01	−0-01 ± 0-011	−0.01 ± 0.01	NS	
	Tmin (min)	96.3 ± 72.8	88.8 ± 67.2	118.8 ± 77.9	126.3 ± 73.9	NS	
**Glucagon**	Fasting values (pmol/L)	5.54 ± 1.50	5.00 ± 2.35	5.83 ± 2.42	4.92 ± 1.74	NS	
	iAUC (0–60 min) (pmol × min/L)	−20.3 ± 78.9	−3.1 ± 64.7	−32.5 ± 36.8	34.4 ± 62.2	NS	
	iAUC (0–180 min) (pmol × min/L)	−154.1 ± 268.1	−9.4 ± 233.6	−102.5 ± 147.7	11.88 ± 170.4	NS	
	iCmax (pmol/L)	1.04 ± 1.14	1.25 ± 0.78	0.75 ± 0.45	1.92 ± 0.97	*p* = 0.011	C vs D: *p* = 0.012
	Tmax (min)	22.5 ± 18.6	31.3 ± 38.1	50.0 ± 39.1	75.0 ± 56.1	*p* = 0.018	A vs D: *p* = 0.082
	iCmin (pmol/L)	−2.00 ± 1.28	−1.54 ± 1.53	−1.75 ± 1.06	1.83 ± 0.86	NS	
	Tmin (min)	52.8 ± 34.1	56.38 ± 52.1	537 ± 52.1	46.3 ± 47.6	NS	

Data are expressed as mean ± SD and reported from baseline (incremental). Statistical tests: Linear mixed-effects modeling followed by Šidak post-hoc test in case of overall significance.

**Table 4:** Assessment of gastrointestinal symptoms.

		Placebo (n = 12)	Xylitol 7 g (n = 12)	Xylitol 17 g (n = 12)	Xylitol 35 g (n = 12)
Abdominal pain	No of subjects with symptom	0	0	0	0
	max severity *	-	-	-	-
Nausea	No of subjects with symptom	0	0	0	0
	max severity	-	-	-	-
Vomiting	No of subjects with symptom	0	0	0	0
	max severity	-	-	-	-
Diarrhea	No of subjects with symptom	0	0	0	0
	max severity	-	-	-	-
Bowel sounds	No of subjects with symptom	5	9	8	9
	max severity	1.4	1.0	1.25	1.0
Bloating	No of subjects with symptom	0	0	1	1
	max severity	-	-	1.0	1.0
Eructation	No of subjects with symptom	0	1	0	0
	max severity	-	1.0	-	-
Flatulence	No of subjects with symptom	0	0	0	0
	max severity	-	-	-	-

* severity score 1 = mild; 2 = severe. Gastrointestinal symptoms were assessed by the use of a checklist. Participants were asked to choose between “no symptom” (0 points), “mild symptoms“ (1 point) and “severe symptoms” (2 points) for each item.

## Data Availability

The data presented in this study are available on request from the corresponding authors.

## Appendix A

Flow diagram
